# Employment of persons with disabilities in the open labour market: A scoping review

**DOI:** 10.4102/ajod.v15i0.1873

**Published:** 2026-05-08

**Authors:** Themba M. Ximba, Hester M. van Biljon, Fasloen Adams, Lana van Niekerk

**Affiliations:** 1 Division Occupational Therapy, Department of Health and Rehabilitation Sciences, Faculty of Medicine and Health Sciences, Stellenbosch University, Cape Town, South Africa

**Keywords:** decent work and economic growth, disability inclusion, supported employment, inclusive recruitment, wage subsidy, representation, work inclusion

## Abstract

**Background:**

Persons with disabilities are often disproportionately placed in part-time roles, confined to low occupational levels and face a heightened risk of job loss perpetuating a cycle of underemployment, leaving many to earn below the official poverty line. Despite progressive South African employment legislations, many companies still do not meet employment targets for employees with disabilities.

**Objectives:**

The scoping review aimed to summarise the literature and synthesise strategies used to employ and retain persons with disabilities in South African open labour market.

**Method:**

Systematic and manual searches of six databases identified relevant primary sources. The first two authors conducted blinded reviews of 2402 titles and abstracts, followed by 482 full-text reviews. Following conflict resolution, 92 sources were included. A content analysis of charted data followed, and the findings were summarised into two categories and 12 codes.

**Results:**

Programmes shown to improve the representation of persons with disabilities were demand-side employment, inclusive recruitment, disability disclosure, reasonable accommodation, employee referral, internships and apprenticeships, return-to-work, supported employment, wage subsidy and partnering with disability organisations. Stakeholder support enhanced implementation and reduced barriers.

**Conclusion:**

Various programmes were adopted to improve the inclusion of employees with disabilities, but their implementation varied across employers, yielding mixed results.

**Contribution:**

The review highlighted the need for policymakers to strengthen enforcement, for employers to foster inclusive workplace practices and for researchers to expand the scope of inquiry to capture broader dimensions of disability employment. Future research could be conducted under different settings to explore the employment of persons with disabilities.

## Introduction

Globally, persons with disabilities remain underrepresented in the open labour market and are more likely to be employed in precarious, part-time and low-paying positions with limited opportunities for career advancement (Pettinicchio & Maroto 2017; Shahidi et al. 2023). Persistent barriers such as stigma, inaccessible workplaces and limited employer awareness continue to undermine meaningful labour market inclusion. As a result, persons with disabilities often face a high risk of job loss and are disproportionately placed in lower occupational levels, often earning below the official poverty line (Lindstrom, Doren & Miesch 2011; Scotch & McConnel 2017). Despite global and national commitments to equality, a substantial gap between policy intentions and employment outcomes persists. The World Bank (2019) has highlighted that persons with disabilities, including those in South Africa, continue to experience systemic exclusion from economic participation.

In South Africa, the Commission of Employment Equity (CEE) reported low equitable representation of persons with disabilities in the open labour market (Department of Labour 2018). Because the low employment rate of persons with disabilities has the potential to restrict the future sustainable economic growth of the country, it has been recognised that multi-pronged strategies are needed. The literature acknowledges the existence of shallow and short-term gains in creating disability-inclusive employment; however, it argues that these may not be sufficient and thus recommends that in-depth approaches and interventions be investigated to create long-term improvements in the disability-inclusive labour market (Shaw et al. 2022).

In South Africa, 1.2% of workers in both the private and public sectors reported having disabilities in 2022 (Department of Employment and Labour 2023). The United Nations Convention on the Rights of Persons with Disabilities defines persons with disabilities as those:

[*W*]ho have long-term physical, mental, intellectual or sensory impairments which in interaction with various barriers may hinder their full and effective participation in society on an equal basis with others. (United Nations 2008:n.p.)

Similarly, South Africa’s *Employment Equity Amendment Act 4 of 2022* (Republic of South Africa 2023) defines persons with disabilities as those:

[*W*]ho have a long-term or recurring physical, mental, intellectual or sensory impairment which, in interaction with various barriers, may substantially limit their prospects of entry into, or advancement in, employment. (p. 2)

South Africa has been applauded for having socioeconomic policies aimed at addressing historical inequalities that emanated from *apartheid*, but it remains among the world’s 10 most unequal countries, including in employment opportunities (Díaz Pabón et al. 2021). A new political era, constitutional rights and progressive legislations do not equate to the realisation of equality and the achievement of socioeconomic transformation (Muswede 2017).

Governments could improve the representation of persons with disabilities in the workplace by increasing their recruitment, enacting policies to enforce basic education and developing occupational capacity (Asmara, Wedadjati & Sa’adah 2022; Kang 2013). In the study by Yazıcı, Şişman and Kocabaş (2011), it was shown that a policy-based quota method was the preferred approach adopted by some large companies to include and employ persons with disabilities in the workforce. Kalargyrou (2014) emphasised that companies that recognise the individual needs of persons with disabilities may have a competitive advantage over those that do not. According to Waxman (2017), inclusion of persons with disabilities required organisations to value diversity and create an accepting culture that embeds disability. Embedding disability in an organisation’s diversity plan demonstrates management’s commitment to creating a disability-inclusive work environment.

The 2015 Disability Statistics Annual Report (Kraus 2015) reported trends that depicted that persons with disabilities in the United States (US) encounter considerable challenges in gaining meaningful employment, and they were paid less than their counterparts. The same issues prevail in South Africa, despite initiatives such as internships and learnerships aimed at including persons with disabilities in paid employment. Internships provide persons with disabilities with opportunities to acquire marketable and competitive skills (Arksey & O’Malley 2005). A South African single case study by Ndzwayiba and Ned (2017) identified that learnerships and internships can redress low employment prospects. Arksey and O’Malley (2005) concur that these programmes provide opportunities to acquire marketable work experience. However, learnerships and internships were criticised for facilitating employment at low occupational levels and in the most basic roles. A longitudinal survey by Rankin, Roberts and Schöer (2014) concluded that completion of a learnership did not translate to better employment opportunities in terms of income or promotion.

Notwithstanding 31 years of democratic governance in South Africa and the enactment of progressive labour legislations designed to facilitate the inclusion of persons with disabilities within the open labour market, they remain significantly under-represented across all sectors and occupational levels (Department of Employment and Labour 2024).

The aim of the scoping review was to summarise and synthesise peer-reviewed and grey literature on strategies used to improve the representation of persons with disabilities in the open labour market. Scoping reviews provide a systematic and transparent approach to clarifying key concepts, examining the breadth of available evidence and identifying research gaps to inform future policy, practice and research (Arksey & O’Malley 2005; Cacchione 2016).

## Methods

### Methodological framework

The scoping review adopted a five-stage process, described by Arksey and O’Malley (2005:22) and outlined below. Stages 3–5 were done using Covidence by Veritas Health Innovation and Mendeley Reference Manager Version 2.134.0. The Preferred Reporting Items for Systematic Reviews and Meta-Analyses (PRISMA)-ScR and JBI guidelines (Joanna Briggs Institute 2020) were followed. The review question was ‘what strategies improve the representation of employees with disabilities in the open labour market?’

#### Search strategy

The keywords and search strings were developed in partnership with a subject librarian at Stellenbosch University. The search string used was (‘disabled persons’ OR handicapped OR disabilit*) AND (‘open labour market*’ OR ‘open labor market*’ OR ‘labor sector*’ OR employment OR employee* OR employer* OR workplace) AND (inclusion OR represent* OR underemploy* OR under-employ* OR ‘return to work’ OR ‘personnel downsizing’) AND (strateg* OR program* OR intervention* OR plan* OR initiative* OR guide*). The search was conducted twice in 3 years using the same search string to include new literature. Grey literature was also considered for inclusion.

The search results from each respective database were imported into Mendeley Reference Manager where the sources were collated, and duplicates identified and removed. The first author conducted hand searches to further retrieve literature not captured in the initial search. Hand searching involved reviewing the reference lists of the included journal articles to ensure relevant literature was included.

#### Databases

The electronic databases Ebscohost, Google Scholar, Emerald, PubMed, ProQuest and Sabinet provided comprehensive coverage of the literature, including the fields of Occupational Science, Human Resource and Diversity and Inclusion. Table 1 shows the results obtained.

**TABLE 1:** Database search results.

Database	Search string	Search results	Search date
PubMed	(‘disabled persons’ OR handicapped OR disabilit*) AND (‘open labour market*’ OR ‘open labor market*’ OR ‘labor sector*’ OR employment OR employee* OR employer* OR workplace) AND (inclusion OR represent* OR underemploy* OR under-employ* OR ‘return to work’ OR ‘personnel downsizing’) AND (strateg* OR program* OR intervention* OR plan* OR initiative* OR guide*) **Filters applied:** 10 years, English, Adolescent: 13–18 years, Adult: 19–44 years, Middle Aged: 45–64 years. Sorted by Best match	459	09 September 2020
	Filters applied: 2020–2023	117	07 April 2023
Ebscohost research databases: Academic search premier (1291) Business source premier (518) CINAHL (254) Africa-Wide information (21)	(‘disabled persons’ OR handicapped OR disabilit*) AND (‘open labour market*’ OR ‘open labor market*’ OR ‘labor sector*’ OR employment OR employee* OR employer* OR workplace) AND (inclusion OR represent* OR underemploy* OR under-employ* OR ‘return to work’ OR ‘personnel downsizing’) AND (strateg* OR program* OR intervention* OR plan* OR initiative* OR guide*) **Limiters** – Published Date: 2010-01-01–2020-12-31; Language: English; Year Published: 2010–2020; Language: English; Age Groups: Adolescent: 13–18 years, Adult: 19–44 years, Middle Aged: 45–64 years; Language: English **Expanders** – Apply equivalent subjects **Search modes** – Boolean/Phrase	2084	09 September 2020
	Published Date: 01 September 2020–31 December 2023	10	06 April 2023

CINAHL, cumulative index to nursing and allied health literature.

#### Selection criteria

The selection criteria were finalised through group discussions among all authors.

**Inclusion criteria:** Sources were included if:

- they contained primary research
- were published in English between January 2010 and April 2023
- pertained to policy developments and emerging employment patterns
- described employment practices or strategies aimed at increasing the representation of persons with disabilities in the open labour market.

Because of the focus on work, the sources included pertained to persons with disabilities in the economically active population, which spans from 15 years to 64 years.

**Exclusion criteria:** Sources were not included if these pertained:

- interventions related to self-employment
- including micro-enterprises and small businesses
- employment outside the open labour market.

Sources that described general disability inclusion strategies or health and employee wellness initiatives were not included, as these fell outside the scope of competitive employment. In competitive employment, jobs are not earmarked for persons with disabilities.

#### Screening and selection

In stage 3, study selection commenced with a review of titles and abstracts, followed by full-texts review. During each of these stages, all the selection criteria provided above were applied. Screening was conducted using Covidence, employing a blind review process to mitigate potential bias during systematic screening. The platform concealed reviewers’ decisions from one another until each reviewer had independently cast their vote. Credibility was established through a dual screening mode, where each source required agreement from two reviewers before proceeding to the next stage of the review (Covidence 2023). Discrepancies were resolved through discussion between the reviewers to reach consensus. Where consensus could not be achieved, the final decision was made by the last author.

#### Data extraction

In stage 4, data charting was done to extract key information including year of publication, authorship, the main conclusions drawn and reported strategies aimed at increasing the representation of persons with disabilities in employment.

#### Data synthesis

A descriptive analytical approach using ATLAS.ti (2023) Version 23.2.1.26990 was used for data analysis. A content analysis was done on the data extracted in Stage 4. A qualitative data analysis strategy by King and Horrocks (2010) was adopted to ensure rigour. The researchers familiarised themselves with the data, identified themes, clustered themes and constructed a summary of the findings. Table 2 depicts the results of categories and codes developed, and supplementary data are available for more details (Online Appendix 1).

**TABLE 2:** Data analysis summary.

Categories	Code
Barriers, challenges, discrimination and exclusion	Distraction to disability development Improve attraction of talent Influence on employees Hesitation to hire persons with disabilities
Programmes improving the representation of persons with disabilities in the≈workplace	Disability disclosure and reasonable accommodation Return-to-work programme Supported employment programme Partner with employment specialists, disability organisations and rehabilitation professionals Demand-side employment and inclusive recruitment Employee referral programme Internship and apprenticeship programmes Wage subsidy programme Individual placement and support (IPS)

Peer debriefing was used to enhance the accuracy of findings and data analysis. This was done to improve trustworthiness and to ensure that readers will resonate with the study (Creswell 2009). Through data triangulation, the analysed data were cross-checked for the confirmability of data and the construction of a coherent justification for themes (Creswell 2009; eds. Sapsford & Jupp 2006). Table 2 reports on the challenges found to hinder the inclusion of persons with disabilities and programmes affecting the representation of employees with disabilities in the competitive labour market.

### Ethical considerations

Ethical clearance to conduct this study was obtained from the Stellenbosch University (SU) the Health Research Ethics Committee (HREC) (No. S20/07/185).

## Review findings

**FIGURE 1:**
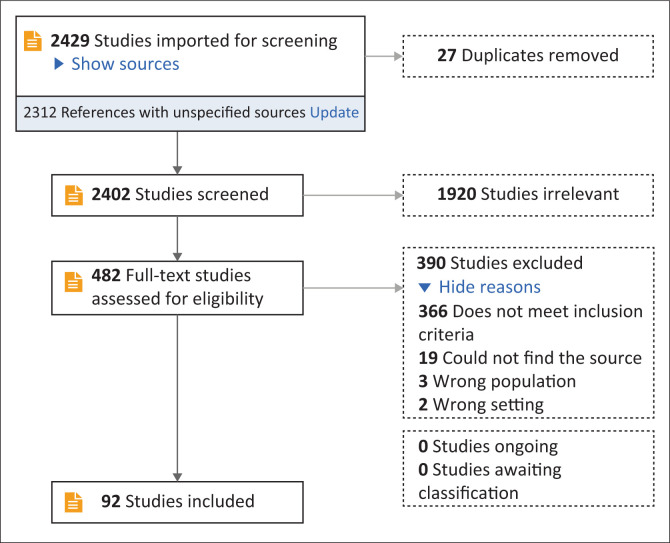
Preferred Reporting Items for Systematic Reviews and Meta-Analyses flow diagram.

Figure 1 contains a PRISMA flow diagram that reports the results obtained for each stage of the process.

Of the 2429 studies imported for screening, Covidence identified 27 duplicates, which were removed. The remaining 2402 studies were screened, and 1920 studies were found to be irrelevant. Of the 482 full-texts reviewed, a further 390 were excluded, leaving 92 sources that were included in the review.

Table 2 depicts the two categories and related codes that emerged during data analysis.

### Barriers, challenges, discrimination and exclusion

Multiple studies (Buhariwala, Wilton & Evans 2015; Nieminen et al. 2012; Teindl et al. 2018) identified stigma, preconceptions and lack of meaningful accommodation as recurring social barriers to sustainable employment for persons with disabilities. The studies concurred that there is a need to redress discrimination and exclusion in employment, as these impede disability inclusion and access to salaried employment in mainstream places of work. This could be done by dispelling disability myths and developing relevant programmes.

According to Bucğīunienże and Kazlauskaitże (2010), factors that restrict the inclusion of employees into the workforce were inability to attract candidates with disabilities, the need for reasonable accommodation and lack of preparation of managers and co-workers. The South African Human Rights Commission (2015) reported that among the barriers that hindered inclusion of employees with disabilities are attitudinal, physical, communication and social barriers. Botha et al. (2023) argued that policymakers and programme designers need to investigate obstacles to transformation in the South African labour market that exclude persons with disabilities.

Findings of a mixed-method sequential transformative study (Sampana & De la Cruz 2020) reported that barriers to inclusion of persons with disabilities were the absence of disability awareness or inclusivity policies in organisations, employers’ perception of limited competence among persons with disabilities and relatively high costs of employment. Conversely, a sense of social belonging was obtained through a good job-match, effective support and when persons with disabilities felt they mattered (Gustafsson, Peralta & Danermark 2018). Sincerely worded diversity statements that did not, in fact, facilitate inclusion led persons with disabilities, as potential job applicants to feel they were not valued (Couture & Johnson 2017). According to Sampana and De la Cruz (2020), potential solutions to barriers were financial support and subsidies, capacity building, partnership with industry and government and enhancement of current non-discriminatory laws. Nieminen et al. (2012) asserted that training interventions or programmes enabled successful integration of persons with disabilities into social inclusion and employment same as the absence thereof hindered their inclusion. The Europe 2020 Strategy lack of explicit reference to disability inclusion, weakened its advocacy for an inclusive society (Choi & Calero 2013). Across organisational and policy levels, the evidence consistently suggests that symbolic commitments to inclusion, in the absence of enforceable strategies and capacity-building interventions, fail to translate into meaningful employment outcomes for persons with disabilities.

Chan et al. (2010) identified that human resource and hiring managers who participated in their quantitative descriptive survey research did not perceive persons with disabilities as reliable and productive members of the workforce. In the qualitative research by Annett (2017), six supervisors interviewed were seen to exaggerate the risk associated with hiring persons with disabilities. The US government survey data by Jasper and Waldhart (2013) reported that employers held varied concerns about hiring of employees with disabilities. In addition, the survey also reported that employers with fewer employees were more hesitant to hire persons with disabilities. Multiple studies highlighted concerns among employers regarding employees with disabilities, including perceptions that they are less reliable or productive, exaggerated views of the risks involved and hesitation to employ them, particularly among smaller employers.

Findings of a quantitative descriptive survey using hierarchical regression analysis reported that disability legislation and training could change hiring attitudes towards persons with disabilities (Chan et al. 2010). Kalargyrou and Volis (2014) argued that a diversity team entrusted with the responsibility to facilitate awareness and inclusion practices helped to identify, hire and retain employees with disabilities by holding educational workshops and sessions that increased the acceptance in the workplace and reduced barriers. Awareness training was shown to encourage employment of persons with disabilities (Moody et al. 2017).

This section demonstrated that barriers to employment are predominantly socially and organisationally produced, thereby positioning employer led intervention as critical leverage points for improving representation. Furthermore, the analysis indicated that the implementation of inclusive recruitment, explicit disability statements, disability awareness initiatives, reasonable accommodation measures and comprehensive inclusivity policies constituted a critical strategy for enhancing employers’ capacity to attract, integrate and retain individuals with disabilities.

### Programmes improving the representation of persons with disabilities in the workplace

The review identified six programmes that addressed the representation of persons with disabilities in the workplace. These were: the disclosure and accommodation of disability, having return-to-work, supported employment (SE) and internship or apprenticeship programmes, the collaboration with employment and rehabilitation specialist and demand side employment.

#### Disability disclosure and reasonable accommodation

Lindsay et al. (2019) asserted that employers hiring candidates with disabilities should provide support to employees that disclose their disability status, provide reasonable accommodation and support them in building trust with their colleagues. Positive reactions by organisations to disability disclosure were shown to foster trust, inclusive decision-making and create psychological safety in the workplace, which increased retention and employees’ sense of work-efficacy (Kirk-Brown & Van Dijk 2014). Reassignment of employees with disabilities to more suitable jobs was reported as a recognised retention strategy by a qualitative study of Roessler et al. (2017). Organisational loyalty and dedication were found to predict organisations’ success in adjusting job designs to meet employees’ needs, providing reasonable accommodations, and create conducive organisational culture among the employees with disabilities and their colleagues (Hashim & Wok 2014). According to Kelly (2012), work adjustments comprised making relevant modifications to work schedules, tasks, jobs and work environments.

#### Return-to-work programme

O’Ferrall and Davey (2016) contended that a rigorous return-to-work plan should include several critical aspects such as a phased return, reasonable accommodations, access to work schemes, regular reviews, disclosure to co-workers, workplace buddy or mentor, disabled employee network and wellness recovery plans. These aspects were deemed necessary for return-to-work initiatives to be a success and lessened feelings of anxiety that employees might have after being away from work. Kelly (2012) argued that a sustainable and successful return-to-work plan incorporated a disability management programme. This was achieved by ensuring that employees were committed and motivated, the plan focused on abilities, had supportive leaders and achieved the removal of barriers. Personalised social support in the work, community and family context was crucial to return-to-work and facilitated balance in everyday work life (Holmlund, Hultling & Asaba 2018). The qualitative multiple case study by Bush et al. (2016) Indicated that conjectures drawn about successful return-to-work recommended involvement in productive activities, maintaining roles with cognitive demands and individualised job adjustments.

#### Supported employment programme

Supported employment, particularly through the evidence-based Individual Placement and Support (IPS) model, has consistently demonstrated strong effectiveness in improving employment outcomes for persons with disabilities, including mental health service users. Supported employment enhances social inclusion by employing a carefully considered job-matching process that promotes social support and integration in workplace environments (Gustafsson et al. 2018). Evidence from specific populations further illustrates its impact; for example, Ottomanelli, Barnett and Goetz (2014) found SE to significantly improve employment outcomes for veterans with spinal cord injuries participating in vocational rehabilitation programmes. More broadly, SE has been shown to improve access to work, increase placement rates and support sustained participation in competitive employment for persons with disabilities, and it is considered one of the most suitable and context-relevant strategies for addressing employment barriers in low- and middle-income countries (Drake & Bond 2023).

Individual Placement and Support, the most rigorously evaluated form of SE, has been validated through more than two dozen randomised controlled trials, demonstrating that participants are more than twice as likely to secure competitive employment compared with individuals receiving traditional vocational rehabilitation services (Bond, Drake & Pogue 2019). In addition to employment benefits, SE also yields positive mental health outcomes, as work is reframed in a meaningful and empowering way (Rødevand et al. 2017). Further evidence highlights that SE interventions are cost-efficient across diverse disability groups (Engelbrecht et al. 2017).

In low- and middle-income countries, SE has proven to be a viable and adaptable strategy although implementation may require adjustments to accommodate contextual realities such as labour market constraints, employer attitudes and resource limitations (Mascayano & Drake 2024; Van Niekerk et al. 2015). Beyond its practical effectiveness, SE also aligns with broader disability-inclusive philosophies. Bell (2020) emphasises that SE facilitates transition into the open labour market in ways that reflect a shift away from the medical model of disability towards a social model that recognises and addresses environmental and systemic barriers to participation. Collectively, the evidence positions SE – especially IPS – as a socially inclusive, adaptable and empirically supported strategy that enhances competitive employment outcomes and promotes occupational justice for persons with disabilities.

#### Partnerships with employment specialists, disability organisations and rehabilitation professionals

Employment specialists were shown to play a crucial role in customising jobs for persons with disabilities, conducting needs analysis, providing training and offering financial support (Riesen & Morgan 2018). In a study by Abma et al. (2013), partnerships between rehabilitation professionals and supervisors were found to be important for effective support of return-to-work. Companies were advised to incorporate disability as a fundamental part of their inclusion and diversity policies, establish partnerships with vocational rehabilitation agencies to recruit suitable qualified candidates and run internship programmes targeting individuals with disabilities (Bezyak et al. 2020).

#### Demand-side employment and inclusive recruitment

Recruitment and hiring practices could be reviewed and audited to comply with accessibility needs of persons with disabilities. A web-based survey of US organisations conducted by Phillips et al. (2019) found that supervisors should be trained in inclusive interviewing and hiring practices, as well as in establishing partnerships with organisations of and for persons with disabilities to support the recruitment of individuals with disabilities. Kalargyrou and Volis (2014) argued that disability awareness, support, coaching, workplace accommodation and proactive recruitment of persons with disabilities proved to diversify the workforce to be inclusive. Employees with disabilities were shown to be loyal, they help create a positive organisational culture and effectively refer their peers with disabilities through employee referral programme (Bucğīunienże & Kazlauskaitże 2010; Kalargyrou 2014).

#### Internship and apprenticeship programmes

A qualitative case study by Kalef, Barrera and Heymann (2014) found that internships provided participants with training, work experience and increased employability and provided job coaches to eliminate possible work challenges that interns could encounter. Apprenticeship programmes for persons with disabilities promoted diversity in the labour market and improved inclusive organisational culture (Berth 2020; Da Silva Chang, Duarte & Veloso 2019). Through the part-time employment and wage subsidy programme, the inclusion of persons with disabilities in the labour market was promoted and employee turnover was reduced, argued Angelov and Eliason (2018).

The assessment of programme effectiveness and implementation revealed mixed success in outcomes across employers. Collectively, these initiatives emerged as critical enablers, they demonstrated progress in fostering inclusivity, prioritised the provision of personalised social support, reasonable accommodations tailored to individual needs and strategic partnerships with disability specialists.

## Implications and recommendations

The scoping review aimed to summarise and synthesise the literature on strategies used to employ and retain persons with disabilities. It applied a methodological framework to identify knowledge gaps, synthesise findings and share existing evidence. The review drew on published and grey literature from low, middle and high-income countries. All sources were published in English between 2010 and 2023 and retrieved from various electronic databases, including Ebscohost, Google Scholar, Emerald, PubMed, ProQuest and Sabinet. The principal author also conducted hand searches on the reference lists of the included journal articles.

For decades of their existence, disability legislations enforce the inclusion of employees with disabilities in the workplaces. Majola and Dhunpath (2016) argued that the attention has been on enacting legislations to overcome barriers without ensuring that employers develop workplace specific disability employment policies. Given the existence of disability inclusion legislations, there is a need to establish programmes that guide and enable employers to increase employment of persons with disabilities (Gupta, Sukhai & Wittich 2021). More research is required to understand issues around disability and employment, stressed Tompa, Samosh and Boucher (2020). This review identified two categories and 13 related codes derived from coding of extracted data using ATLAS.ti. Firstly, it outlines the challenges hindering the inclusion of persons with disabilities, while secondly, it highlights programmes improving their representation in the open labour market.

Firstly, the category discussed barriers, challenges, discrimination and exclusion of persons with disabilities with four related codes highlighting disability development, better attraction of talent, positively influencing employees and addressing employers’ hesitation to hire persons with disabilities. Employees with disabilities were at risk of being stagnant in their careers, and their barriers were likely to persist rather than be addressed. A qualitative study conducted in Georgia by Verulava and Bedianashvili (2021) mirrored review findings that barriers included lack of effective legislation regulating the employment of persons with disabilities, negative perception about the competencies and skills of persons with disabilities, lack of information on available vacancies or information in inaccessible formats, lack of development of inclusive education resulting in persons with disabilities not attaining higher education, inaccessible physical environment and rejection of persons with disabilities by their societies. The removal of these barriers by employers and relevant stakeholders could change the work culture and notably improve the employment prospects of employees with disabilities (Chumo, Kabaria & Mberu 2023).

Secondly, the category discussed programmes affecting the representation in the workplace. The scoping review identified nine strategies that were used globally to employ and retain persons with disabilities. Evidence indicated that programmes like internships and learnerships primarily resulted in temporal inclusion without structural transformation, increasing short-term representation of persons with disabilities in entry-level roles, without enabling sustained career progression. Similarly, Majola and Dhunpath (2016) asserted that switchboard, clerical or similar lower-level vacant positions were specifically earmarked for recruiting candidates with disabilities in the South African public service.

Studies in this review indicate that employers implement various programmes to hire persons with disabilities. However, these programmes encountered challenges when attempting to address under-representation of persons with disabilities in the open labour market. In the absence of substantial evidence on effective strategies to improve the employability of persons with disabilities, the need for research was identified.

The identified barriers undermined the success and effectiveness of programmes, thereby limited the inclusion of persons with disabilities in the open labour market. To address this, organisations are advised to adopt strategies to position themselves as employers of choice for candidates with disabilities and to foster a disability-confident culture. This review aimed to advance theoretical discourse on disability-inclusive employment by applying the analysis within the social model of disability. Consistent with the premises of the social model of disability, the review identified a range of attitudinal, structural, communication and social barriers that continue to restrict inclusion. These barriers manifest through stigma, entrenched misconceptions, limited disability awareness, ineffective or poorly implemented inclusion policies and the inadequate provision of meaningful, reasonable accommodations (South African Human Rights Commission 2015).

### Contributions

#### Theoretical contribution

The review contributes conceptually by highlighting the key challenges that hinder the labour-market inclusion of persons with disabilities and by mapping the types of interventions that can facilitate their recruitment into competitive employment.

#### Practical contribution

The review provides practical insights by calling on employers to actively remove workplace barriers and cultivate inclusive, supportive environments that enable persons with disabilities to participate fully and productively.

#### Policy contribution

The review reinforces the imperative for policymakers to rigorously enforce and monitor disability policies and legislation to promote fair and equitable labour-market participation for persons with disabilities.

### Limitations

Substantial methodological heterogeneity and a pronounced risk of bias concerning non-English-language literature affected the depth and scope of findings presented in the review. However, synthesised evidence from diverse research designs might mitigate publication bias. The comprehensive search was repeated in an attempt to include significant contributions related to barriers and programmes influencing the representation of persons with disabilities in the open labour market.

## Conclusion

This scoping review synthesised existing literature on strategies used to recruit and retain persons with disabilities in employment. Using systematic and manual search methods, 92 sources were included from an initial pool of 2402 sources screened at the title and abstract stage.

The findings indicate that South Africa, similar to many other countries, has progressive legislative frameworks that promote the inclusion of persons with disabilities in the open labour market. Despite these policies, employers continue to fall short of disability employment targets, resulting in the persistent underrepresentation of persons with disabilities. Where employed, individuals with disabilities are often concentrated in precarious positions, encounter systemic and attitudinal barriers, face heightened risks of job insecurity and experience limited opportunities for career progression.

Several initiatives were identified as having potential to improve the representation of persons with disabilities in competitive employment. These included demand-side employment strategies, inclusive recruitment practices, disability disclosure, the provision of reasonable accommodations, employee referral programmes, internship and partnerships with disability organisations. However, significant variability in the implementation of these initiatives across organisations yielded mixed successes.

Overall, this scoping review highlights a substantial gap between disability-inclusive legislations and their practical implementation within workplaces. Strengthening compliance with existing disability policies and improving employer accountability may be critical to advancing inclusive employment practices and reducing the ongoing exclusion of persons with disabilities from meaningful and sustainable employment.

## References

[CIT0001] Abma, F.I., Bültmann, U., Varekamp, I. & Van der Klink, J.J.L., 2013, ‘Workers with health problems: Three perspectives on functioning at work’, *Disability and Rehabilitation* 35(1), 20–26. 10.3109/09638288.2012.687027 22620284

[CIT0002] Angelov, N. & Eliason, M., 2018, ‘Wage subsidies targeted to jobseekers with disabilities: Subsequent employment and disability retirement’, *IZA Journal of Labor Policy* 7(1), 12. 10.1186/s40173-018-0105-9

[CIT0003] Annett, M., 2017, ‘Categorizing supervisor reflections on risks of hiring persons with disabilities’, *Journal of Organizational Psychology* 17(4), 29–35, viewed 05 February 2021, from http://www.na-businesspress.com/JOP/AnnettM_17_4_.pdf.

[CIT0004] Arksey, H. & O’Malley, L., 2005, ‘Scoping studies: Towards a methodological framework’, *International Journal of Social Research Methodology: Theory and Practice* 8(1), 19–32. 10.1080/1364557032000119616

[CIT0005] Asmara, A.F., Wedadjati, R.S. & Sa’adah, N., 2022, ‘Evaluating equal employment opportunity in Indonesian industries to accommodate disabled workers’, *International Journal of Business and Systems Research* 16(5/6), 624–643. 10.1504/ijbsr.2022.10039574

[CIT0006] ATLAS.ti, 2023, *The Ultimate Guide to Qualitative Research - Part 1: The Basics*, viewed 09 November 2023, from https://atlasti.com/guides/qualitative-research-guide-part-1/qualitative-research.

[CIT0007] Bell, M., 2020, ‘People with intellectual disabilities and labour market inclusion: What role for EU labour law?’, *European Labour Law Journal*, viewed 26 February 2026, from https://papers.ssrn.com/sol3/papers.cfm?abstract_id=3502855.

[CIT0008] Berth, R., 2020, ‘In pursuit of inclusivity: Working with apprentices with disabilities’, *Benefits Magazine* 57(3), 20–25, viewed from http://search.ebscohost.com/login.aspx?direct=true&db=buh&AN=141856239&site=ehost-live&scope=site.

[CIT0009] Bezyak, J., Moser, E., Iwanaga, K., Wu, J.-R., Chen, X. & Chan, F., 2020, ‘Disability inclusion strategies: An exploratory study’, *Journal of Vocational Rehabilitation* 53(2), 183–188. 10.3233/JVR-201095

[CIT0010] Bond, G.R., Drake, R.E. & Pogue, J.A., 2019, ‘Expanding individual placement and support to populations with conditions and disorders other than serious mental illness’, *Psychiatric Services* 70(6), 488–498. 10.1176/appi.ps.201800464 30813865

[CIT0011] Botha, M., Fischer Mogensen, K., Ebrahim, A. & Brand, D., 2023, ‘In search of a landing place for persons with disabilities: A critique of South Africa’s skills development programme’, *International Journal of Discrimination and the Law* 23(1–2), 163–180. 10.1177/13582291231162315

[CIT0012] Bucğīunienże, I. & Kazlauskaitże, R., 2010, ‘Integrating people with disability into the workforce: The case of a retail chain’, *Equality, Diversity and Inclusion: An International Journal* 29(5), 534–538. 10.1108/02610151011052816

[CIT0013] Buhariwala, P., Wilton, R. & Evans, J., 2015, ‘Social enterprises as enabling workplaces for people with psychiatric disabilities’, *Disability & Society* 30(6), 865–879. 10.1080/09687599.2015.1057318

[CIT0014] Bush, E.J., Hux, K., Guetterman, T.C. & McKelvey, M., 2016, ‘The diverse vocational experiences of five individuals returning to work after severe brain injury: A qualitative inquiry’, *Brain Injury* 30(4), 422–436. 10.3109/02699052.2015.1131849 26910611

[CIT0015] Cacchione, P.Z., 2016, ‘The evolving methodology of scoping reviews’, *Clinical Nursing Research* 25(2), 115–119. 10.1177/1054773816637493 26976609

[CIT0016] Chan, F., Strauser, D., Maher, P., Lee, E.-J., Jones, R. & Johnson, E.T., 2010, ‘Demand-side factors related to employment of people with disabilities: A survey of employers in the Midwest region of the United States’, *Journal of Occupational Rehabilitation* 20(4), 412–419. 10.1007/s10926-010-9252-6 20602153

[CIT0017] Choi, A. & Calero, J., 2013, ‘The contribution of the population of disabled people to the attainment of Europe 2020 strategy headline targets’, *Disability & Society* 28(6), 853–873. 10.1080/09687599.2013.808573

[CIT0018] Chumo, I., Kabaria, C. & Mberu, B., 2023, ‘Social inclusion of persons with disability in employment: What would it take to socially support employed persons with disability in the labor market?’, *Frontiers in Rehabilitation Sciences* 4, 1125129. 10.3389/fresc.2023.1125129 37456796 PMC10349392

[CIT0019] Couture, K.A. & Johnson, K.R., 2017, ‘Website barriers to employment for people with disabilities’, *Journal of Business Diversity* 17(1), 110–121, viewed n.d., from http://www.na-businesspress.com/JBD/CoutureKA_Web17_1_.pdf.

[CIT0020] Covidence, 2023, *Screening by title and abstract in Covidence*, viewed 08 November 2023, from https://support.covidence.org/help/screening-by-title-and-abstract.

[CIT0021] Creswell, J.W., 2009, *Research design: Qualitative, quantitative, and mixed methods approaches*, 3rd edn., Sage, Los Angeles.

[CIT0022] Da Silva Chang, S.R., Duarte, M.M.N.B. & Veloso, J.R.P., 2019, ‘Paths, misplacements and challenges in Brazilian VET for people with disability’, *Journal of Vocational Education & Training* 71(3), 368–384. 10.1080/13636820.2019.1623296

[CIT0023] Department of Employment and Labour, 2023, *23rd commission for employment equity (CEE) annual report 2022–2023*, viewed 16 July 2023, from https://www.labour.gov.za/DocumentCenter/Reports/Annual%20Reports/Employment%20Equity/2022-2023/23rd%20Annual%20CEE%20Report.pdf.

[CIT0024] Department of Employment and Labour, 2024, *24th commission for employment equity (CEE) annual report (2023/24)*, viewed 03 December 2025, from https://www.labour.gov.za/DocumentCenter/Reports/Annual%20Reports/Employment%20Equity/2024/24th%20Commission%20for%20Employment%20Equity%20Annual%20Report.pdf.

[CIT0025] Department of Labour, 2018, *18th commission for employment equity: Annual report 2017–2018*, Department of Labour, Pretoria.

[CIT0026] Díaz Pabón, F.A., Leibbrandt, M., Ranchhod, V. & Savage, M., 2021, ‘Piketty comes to South Africa’, *British Journal of Sociology* 72(1), 106–124. 10.1111/1468-4446.12808 33764517

[CIT0027] Drake, R.E. & Bond, G.R., 2023, ‘Individual placement and support: History, current status, and future directions’, *Psychiatry and Clinical Neurosciences Reports* 2(3), e122. 10.1002/pcn5.122 38867819 PMC11114326

[CIT0028] Engelbrecht, M., van Niekerk, L., Coetzee, Z. & Hajwani, Z., 2017, ‘Supported Employment for people with mental disabilities in South Africa: Cost calculation of service utilisation’, *South African Journal of Occupational Therapy* 47(2), 11–16. 10.17159/231-3833/1017/v47n2a3

[CIT0029] Gupta, S., Sukhai, M. & Wittich, W., 2021, ‘Employment outcomes and experiences of people with seeing disability in Canada: An analysis of the Canadian survey on disability 2017’, *PLoS One* 16(11), e0260160. 10.1371/journal.pone.0260160 34843553 PMC8629233

[CIT0030] Gustafsson, J., Peralta, J. & Danermark, B., 2018, ‘Supported employment and social inclusion – Experiences of workers with disabilities in wage subsidized employment in Sweden’, *Scandinavian Journal of Disability Research* 20(1), 26–36. 10.16993/sjdr.36

[CIT0031] Hashim, J. & Wok, S., 2014, ‘Predictors to employees with disabilities’ organisational behaviour and involvement in employment’, *Equality, Diversity and Inclusion: An International Journal* 33(2), 193–209. 10.1108/EDI-03-2012-0018

[CIT0032] Holmlund, L., Hultling, C. & Asaba, E., 2018, ‘Mapping out one’s own paths toward work: Focus on experiences of return to work after spinal cord injury’, *Qualitative Health Research* 28(13), 2020–2032. 10.1177/1049732318782706 29911499

[CIT0033] Jasper, C.R. & Waldhart, P., 2013, ‘Employer attitudes on hiring employees with disabilities in the leisure and hospitality industry: Practical and theoretical implications’, *International Journal of Contemporary Hospitality Management* 25(4), 577–594. 10.1108/09596111311322934

[CIT0034] Joanna Briggs Institute, 2020, *JBI manual for evidence synthesis – 3.2.8 data extraction*, JBI, viewed 30 September 2020, from https://jbi-global-wiki.refined.site/space/MANUAL/355863557/Previous+versions?attachment=https://jbi-global-wiki.refined.site/download/attachments/355863557/JBI_Reviewers_Manual_2020June.pdf&type=application/pdf&filename=JBI_Reviewers_Manual_2020June.pdf.

[CIT0035] Kalargyrou, V., 2014, ‘Gaining a competitive advantage with disability inclusion initiatives’, *Journal of Human Resources in Hospitality & Tourism* 13(2), 120–145. 10.1080/15332845.2014.847300

[CIT0036] Kalargyrou, V. & Volis, A.A., 2014, ‘Disability inclusion initiatives in the hospitality industry: An exploratory study of industry leaders’, *Journal of Human Resources in Hospitality & Tourism* 13(4), 430–454. 10.1080/15332845.2014.903152

[CIT0037] Kalef, L., Barrera, M. & Heymann, J., 2014, ‘Developing inclusive employment: Lessons from Telenor Open Mind’, *WORK: A Journal of Prevention, Assessment & Rehabilitation* 48(3), 423–434. 10.3233/WOR-131783 24284678

[CIT0038] Kang, D., 2013, ‘Why would companies not employ people with disabilities in Korea?’, *Asia Pacific Journal of Social Work and Development* 23(3), 222–229. 10.1080/02185385.2013.818202

[CIT0039] Kelly, A., 2012, ‘Strategies for a successful return to work’, *Plans and Trusts* 30(6), 9–12.

[CIT0040] King, N. & Horrocks, C., 2010, *Interviews in qualitative research*, Sage, Los Angeles.

[CIT0041] Kirk-Brown, A.K. & Van Dijk, P.A., 2014, ‘An empowerment model of workplace support following disclosure, for people with MS’, *Multiple Sclerosis Journal* 20(12), 1624–1632. 10.1177/1352458514525869 24619936

[CIT0042] Kraus, L., 2015, *2015 disability statistics annual report*, Univeristy of New Hampshire, Durham.

[CIT0043] Lindsay, S., Cagliostro, E., Leck, J., Shen, W. & Stinson, J., 2019, ‘Employers’ perspectives of including young people with disabilities in the workforce, disability disclosure and providing accommodations’, *Journal of Vocational Rehabilitation* 50(2), 141–156. 10.3233/JVR-180996

[CIT0044] Lindstrom, L., Doren, B. & Miesch, J., 2011, ‘Waging a living: Career development and long-term employment outcomes for young adults with disabilities’, *Exceptional Children* 77(4), 423–434. 10.1177/001440291107700403

[CIT0045] Majola, B.K. & Dhunpath, R., 2016, ‘The development of disability-related employment policies in the South African public service’, *Problems and Perspectives in Management* 14(1), 150–159. 10.21511/ppm.14(1-1).2016.02

[CIT0046] Mascayano, F. & Drake, R.E., 2024, ‘Supported employment as a global mental health intervention’, *Cambridge Prisms: Global Mental Health* 11(e102) 1–6. 10.1017/gmh.2024.112. PMC11504922 39464548

[CIT0047] Moody, L., Saunders, J., Leber, M., Wójcik-Augustyniak, M., Szajczyk, M. & Rebernik, N., 2017, ‘An exploratory study of barriers to inclusion in the European workplace’, *Disability and Rehabilitation* 39(20), 2047–2054. 10.1080/09638288.2016.1217072 27665671

[CIT0048] Muswede, T., 2017, ‘Colonial legacies and the decolonisation discourse in post-apartheid South Africa: A reflective analysis of student activism in higher education’, *African Journal of Public Affairs* 9(5), 200–210.

[CIT0049] Ndzwayiba, N. & Ned, L., 2017, ‘The complexity of disability inclusion in the workplace: A South African study’, in B.M. Altman (ed.), *Factors in studying employment for persons with disability: How the picture can change*, pp. 127–154, Emerald Publishing Limited, Leeds.

[CIT0050] Nieminen, I., Ramon, S., Dawson, I., Flores, P., Leahy, E., Pedersen, M.L. et al., 2012, ‘Experiences of social inclusion and employment of mental health service users in a European union project’, *International Journal of Mental Health* 41(4), 3–23. 10.2753/IMH0020-7411410401

[CIT0051] O’Ferrall, S.M. & Davey, P., 2016, ‘Managing absence: the return to work’, *Occupational Health & Wellbeing* 68(3), 24.

[CIT0052] Ottomanelli, L., Barnett, S.D. & Goetz, L.L., 2014, ‘Effectiveness of supported employment for veterans with spinal cord injury: 2-year results’, *Archives of Physical Medicine and Rehabilitation* 95(4), 784–790. 10.1016/j.apmr.2013.11.012 24316325

[CIT0053] Pettinicchio, D. & Maroto, M., 2017, ‘Employment outcomes among men and women with disabilities: How the intersection of gender and disability status shapes labor market inequality’, in B.M. Altman (ed.), *Factors in studying employment for persons with disability*, pp. 3–33, Emerald Publishing Limited, Bingley.

[CIT0054] Phillips, K.G., Houtenville, A.J., O’Neill, J. & Katz, E., 2019, ‘The effectiveness of employer practices to recruit, hire, and retain employees with disabilities: Supervisor perspectives’, *Journal of Vocational Rehabilitation* 51(3), 339–353. 10.3233/JVR-191050

[CIT0055] Rankin, N., Roberts, G. & Schöer, V., 2014, *The success of learnerships? lessons from South Africa’s training and education programme*, WIDER, Helsinki.

[CIT0056] Republic of South Africa (2023). *Employment Equity Amendment Act 4 of 2022*, Pretoria, viewed 17 July 2025, from https://www.gov.za/sites/default/files/gcis_document/202304/48418employment-equity-amendment-act42022.pdf.

[CIT0057] Riesen, T. & Morgan, R.L., 2018, ‘Employer views of customized employment: A focus group analysis’, *Journal of Vocational Rehabilitation* 49(1), 33–44. 10.3233/JVR-180952

[CIT0058] Rødevand, L., Ljosaa, T.M., Granan, L.P., Knutzen, T., Jacobsen, H.B. & Reme, S.E., 2017, ‘A pilot study of the individual placement and support model for patients with chronic pain’, *BMC Musculoskeletal Disorders* 18(1), 550. 10.1186/s12891-017-1908-3 29282028 PMC5746000

[CIT0059] Roessler, R.T., Rumrill, P.D. & Timblin, R.I., 2017, ‘Focus group perspectives on high-priority employment barriers facing Americans with multiple sclerosis’, *Journal of Vocational Rehabilitation* 47(2), 223–233. 10.3233/JVR-170897

[CIT0060] Sampana, E.Y. & De la Cruz, A., 2020, ‘Count them in! Inclusion of persons with disabilities in a diversified workforce: A transformative mixed-methods study’, *Journal of Management and Training for Industries* 7(2), 26–60. 10.12792/JMTI.7.2.26

[CIT0061] Sapsford, R. & Jupp, V. (eds.), 2006, *Data collection and analysis*, 2nd edn., SAGE Publications in Association with the Open University, London.

[CIT0062] Scotch, R.K. & McConnel, C.E., 2017, ‘Disability and the future of work: A speculative essay’, in B.M. Altman (ed.), *Factors in studying employment for persons with disability*, pp. 249–266, Emerald Publishing Limited, Bingley.

[CIT0063] Shahidi, F.V., Jetha, A., Kristman, V., Smith, P.M. & Gignac, M.A.M., 2023, ‘The employment quality of persons with disabilities: Findings from a national survey’, *Journal of Occupational Rehabilitation* 33, 785–795. 10.1007/s10926-023-10113-7 37043125 PMC10090748

[CIT0064] Shaw, J., Wickenden, M., Thompson, S. & Mader, P., 2022, ‘Achieving disability inclusive employment – Are the current approaches deep enough?’, *Journal of International Development* 34(5), 942–963. 10.1002/jid.3692

[CIT0065] South African Human Rights Commission, 2015, *Disability toolkit for the private sector*, South African Human Rights Commission, Braamfontein.

[CIT0066] Teindl, K., Thompson-Hodgetts, S., Rashid, M. & Nicholas, D.B., 2018, ‘Does visibility of disability influence employment opportunities and outcomes? A thematic analysis of multi-stakeholder perspectives’, *Journal of Vocational Rehabilitation* 49(3), 367–377. 10.3233/JVR-180980

[CIT0067] Tompa, E., Samosh, D. & Boucher, N., 2020, *Skill gaps, underemployment and equity of labour-market opportunities for persons with disabilities in Canada*, Public Policy Forum, Ottawa.

[CIT0068] United Nations, 2008, *Article 1 – Purpose | United nations enable*, United Nations Convention on the Rights of Persons with Disabilities, viewed 22 February 2024, from https://www.un.org/development/desa/disabilities/convention-on-the-rights-of-persons-with-disabilities/article-1-purpose.html.

[CIT0069] Van Niekerk, L., Coetzee, Z., Engelbrecht, M., Hajwani, Z. & Terreblanche, S., 2015, ‘Time utilisation trends of supported employment services by persons with mental disability in South Africa’, *WORK: A Journal of Prevention, Assessment & Rehabilitation* 52(4), 825–833. 10.3233/WOR-152149 26409392

[CIT0070] Verulava, T. & Bedianashvili, G., 2021, ‘Work inclusion of persons with disabilities: Employers’ perspectives’, *Quality – Access to Success* 22(182), 159–163, viewed from https://www.researchgate.net/publication/351547400.

[CIT0071] Waxman, D., 2017, ‘Model of successful corporate culture change integrating employees with disabilities’, in B.M. Altman (ed.), *Factors in studying employment for persons with disability*, pp. 155–180, Emerald Publishing Limited, Bingley.

[CIT0072] World Bank, 2019, *Disability inclusion overview*, World Bank, Washington, DC.

[CIT0073] Yazıcı, B., Şişman, Y. & Kocabaş, F., 2011, ‘Determining the problems of disabled employees: A survey study conducted in Eskişehir, Turkey’, *Disability & Society* 26(3), 285–292. 10.1080/09687599.2011.560373

